# Antioxidant and Anti-Inflammatory Effects of Vanillic Acid in Human Plasma, Human Neutrophils, and Non-Cellular Models In Vitro

**DOI:** 10.3390/molecules30030467

**Published:** 2025-01-22

**Authors:** Anna Magiera, Joanna Kołodziejczyk-Czepas, Monika Anna Olszewska

**Affiliations:** 1 Department of Pharmacognosy, Faculty of Pharmacy, Medical University of Lodz, 1 Muszynskiego St., 90-151 Lodz, Poland; anna.magiera@umed.lodz.pl; 2 Department of General Biochemistry, Faculty of Biology and Environmental Protection, University of Lodz, 141/143 Pomorska, 90-236 Lodz, Poland; joanna.kolodziejczyk@biol.uni.lodz.pl

**Keywords:** 4-hydroxy-3-methoxy benzoic acid, neutrophils, PBMCs, plasma protection, protein glycation

## Abstract

Vanillic acid (VA) is a dietary benzoic acid derivative, flavoring agent, and food stabilizer. In this study, the antioxidant and anti-inflammatory potential of VA was explored in vitro and ex vivo in human immune cells and non-cellular models. In neutrophils, VA significantly downregulated the *f*MLP-induced oxidative burst and the generation of reactive oxygen species (ROS); it also suppressed the release of pro-inflammatory cytokines (TNF-α, IL-8) and the tissue-remodeling enzyme elastase-2 (ELA-2) in cells stimulated with LPS and *f*MLP+cytochalasin B. Additionally, VA showed good biocompatibility with human neutrophils and peripheral blood mononuclear cells (PBMCs) across the tested concentrations of 1–50 µg/mL. Furthermore, VA at 1–5 μg/mL enhanced the non-enzymatic antioxidant capacity of human plasma (NEAC) and prevented oxidative and nitrative damage to plasma proteins by protecting tyrosine moieties and thiols from peroxynitrite. VA also inhibited lipid peroxidation and the formation of thiobarbituric acid-reactive substances (at 50 μg/mL) and protein-bound carbonyls (at 5–50 μg/mL) in peroxynitrite-treated plasma. In non-cellular tests, VA acted as a hypochlorous acid and hydrogen peroxide scavenger and inhibited non-enzymatic protein glycation, outperforming the references Trolox and aminoguanidine. Along with existing data from animal models and studies on polyphenol intake, these results might support the synergic role of VA in dietary protection against chronic diseases related to oxidative stress and inflammation.

## 1. Introduction

**Figure 1:**
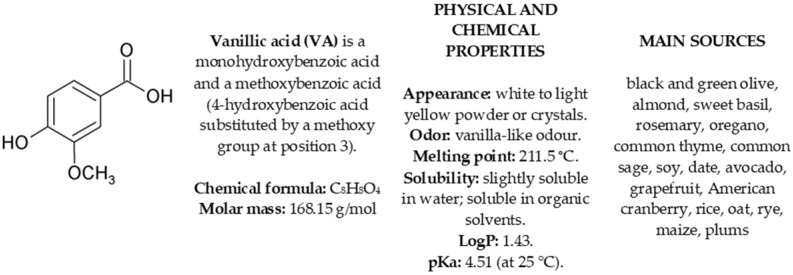
Vanillic acid (VA)—definition, physical and chemical properties and main sources.

Vanillic acid (4-hydroxy-3-methoxy benzoic acid, VA) (Figure 1) is a phenolic compound commonly found in free and conjugated forms in various medicinal herbs and plant-based foods and beverages consumed on a regular daily basis, like fruits, vegetables, grains, teas, juices, wines, and beers [1,2,3,4,5,6,7,8]. The richest known dietary sources of VA (5–14 mg/100 g fw) are dried sage and rosemary leaves, thyme herb, oregano herb, sweet basil, dates, cranberries, olives, and plums [8].

VA is biosynthesized in plants via the shikimic acid pathway. It is also formed as a by-product from the oxidation or degradation of various low- and high-molecular-weight polyphenolic precursors, like vanillin, ferulic acid, or lignin, during plant drying and thermal processing. With its acidic and aromatic properties, VA plays a vital role in sensory characteristics and food preservation by modifying pH and contributing to a sweet, vanilla-like aroma of food products. For these reasons, VA is also manufactured through chemical synthesis. Similar to its corresponding aldehyde, vanillin, which has even stronger aromatic properties and is one of the most significant food additives, VA is used as both a flavoring and stabilizing agent in the food industry [9]. Moreover, VA is a metabolite formed during the digestion of dietary polyphenols after their oral intake, for example, anthocyanins [10,11,12].

Phenolic acids are multifunctional dietary bioactive compounds with significant potential in preventing and managing chronic human diseases. The health-promoting properties of phenolic acids are usually attributed to their relatively high bioavailability after oral administration and wide-ranging antioxidant, anti-inflammatory, and anti-aging effects [13]. Due to the phenolic acid structure and prevalence in food, the role of VA in human health and its therapeutic potential have recently gained increased attention [1]. Numerous human intervention studies have lent weight to the systemic activity of VA by evidencing that it is absorbable from food matrices and circulates in both free and conjugated forms in the human blood after oral intake of polyphenol-rich diets [11,14,15]. Although the pharmacological effects of VA have not been explored as extensively as other dietary prevalent phenolic acids [1,13], the accumulated data from in vivo animal studies support notable bioactivity of VA against disorders related to inflammation and oxidative stress, such as diabetes, osteoarthritis, dyslipidemia, cardiovascular, hepatic, upper respiratory tract, and neurodegenerative diseases [16,17,18,19,20,21,22,23,24]. For instance, the oral administration of VA reduced lipid peroxidation and increased cardiac antioxidants in isoproterenol-induced cardiotoxic rats [24]; normalized the levels of lipids and lipoproteins and upregulated the expression of eNOS in NO-deficient rats [22]; ameliorated hyperglycemia-induced oxidative stress and inflammation in streptozotocin-induced diabetic rats [20]; reduced asthmatic lung inflammation, inhibited lipid peroxidation, and maintained antioxidants enzymes in ovalbumin-induced asthma model in rats [17]; and reduced cartilage destruction and related pain in osteoarthritis rats [19,23]. Some of the molecular pathways and mechanisms associated with these in vivo effects have also been tested in vitro in numerous cellular models, mostly involving animal and cancer cell lines, like, e.g., mouse peritoneal and RAW 264.7 macrophages, Neuro-2α mouse neuroblasts, 3T3-L1 mouse preadipocytes, and Hep3B/HepG2 human hepatic cancer cells [1]. Thus, there remains a notable gap in knowledge regarding the influence of VA on normal human immune cells, such as neutrophils, the crucial players in innate human immunity, contributing significantly to the pro-inflammatory and pro-oxidant pathology of chronic diseases [25]. Additionally, certain other factors that may affect the health benefits of VA are also overlooked. These include its interactions with human proteins and lipids under conditions of oxidative and inflammatory stress, scavenging activity toward various physiologically significant oxidants, and influence on non-enzymatic protein glycation, which is one of the most essential stress and risk factors in the development of diabetes and its complications.

Consequently, the present study aimed to evaluate for the first time the impact of VA on the pro-oxidant and pro-inflammatory functions of human neutrophils ex vivo. The research focused on the release of reactive oxygen species (ROS) during oxidative burst, the secretion of critical cytokines (IL-8 and TNF-α), and an enzyme involved in extracellular matrix remodeling (neutrophil elastase, ELA-2). Moreover, the cellular biocompatibility of VA with human immune cells was assessed in models of neutrophils and peripheral blood mononuclear cells (PBMCs) ex vivo. Furthermore, the protective effects of VA against oxidative and nitrative damage of human plasma components (proteins and lipids) and its influence on the non-enzymatic antioxidant capacity of plasma (NEAC) under oxidative stress conditions were examined. In the last step, the antiglycation properties of VA were tested in a non-cellular in vitro model of hyperglycemic conditions, and the ROS-scavenging profile of VA was characterized in vitro using a panel of reactive oxygen and nitrogen species typical for neutrophil overactivation-related systemic oxidative stress.

## 2. Results and Discussion

### 2.1. Influence on Human Neutrophils Ex Vivo

Neutrophils are key immune cells and primary leukocytes in humans, playing a vital role in defending against pathogens. They can also be activated by various chronic disease risk factors like hyperlipidemia or hyperglycemia, which upregulate extracellular ROS generation, cytokine release, and the formation of neutrophil extracellular traps (NETs) in the cells [25]. This hyperactivation results in persistent oxidative/nitrative stress and inflammation, contributing to tissue damage complications associated with chronic disorders [26,27]. As there is no information on the influence of VA on human neutrophils, the present study evaluated its effects on their pro-oxidant and pro-inflammatory functions in an ex vivo model.

#### 2.1.1. Modulation of Neutrophil Functions—Antioxidant Effects

**Figure 2:**
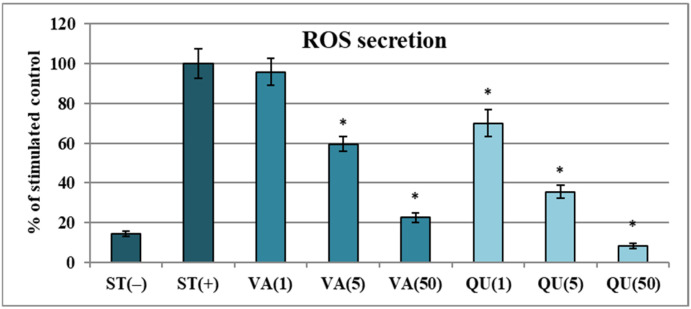
The effect of vanillic acid (VA) at concentrations of 1–50 µg/mL on ROS production by *f*MLP-stimulated neutrophils [%]. The presented data are means ± SD of three independent experiments performed with cells isolated from five independent donors. Positive control: quercetin (QU) at 1–50 µg/mL. Statistical significance of differences (*) was established by ANOVA with a post hoc Dunnett’s test, with * *p* < 0.05 compared with the stimulated control.

In the first step we examined how VA affects the levels of ROS released during respiratory burst of human neutrophils, which is the initial response of immune cells to soluble agonists that triggers ROS production and pro-inflammatory signaling [28]. Quercetin (QU), a potent plant-derived antioxidant with a well-confirmed cellular mechanism of action, served as a positive control. As shown in Figure 2, stimulation of the cells by *f*MLP, a potent chemoattractant derived from bacteria, led to an over 5-fold increase in extracellular ROS generation in activated neutrophils. The process was significantly downregulated by VA at concentrations ranging from 5 to 50 µg/mL (*p* < 0.05), with inhibition rates of 40.5% at 5 µg/mL (29.7 μM) and 77.8% at 50 µg/mL. Notably, ROS secretion at 50 µg/mL of VA did not differ significantly from that measured for control, non-stimulated neutrophils (*p* > 0.05).

Moreover, while the effects of QU were more pronounced due to its structural advantages over the VA molecule, it is noteworthy that the difference in effectiveness between the two compounds at the same concentrations was only 2–3 fold, highlighting the significant antioxidant potential of VA in normal human immune cells. This finding might be supported by comparing the potency of VA to chlorogenic acid, a caffeoylquinic acid, which we tested previously in the same experimental model [6]. Chlorogenic acid is one of the most widespread dietary phenolic acids recognized for its antioxidant capacity-related beneficial health effects in humans. Accordingly, VA at 5 µg/mL appears to be only 1.7 times less capable of limiting the ROS levels in neutrophils than chlorogenic acid, while the effects of both compounds were similar at 50 µg/mL (*p* < 0.05).

#### 2.1.2. Modulation of Neutrophil Functions—Anti-Inflammatory Effects

**Figure 3:**
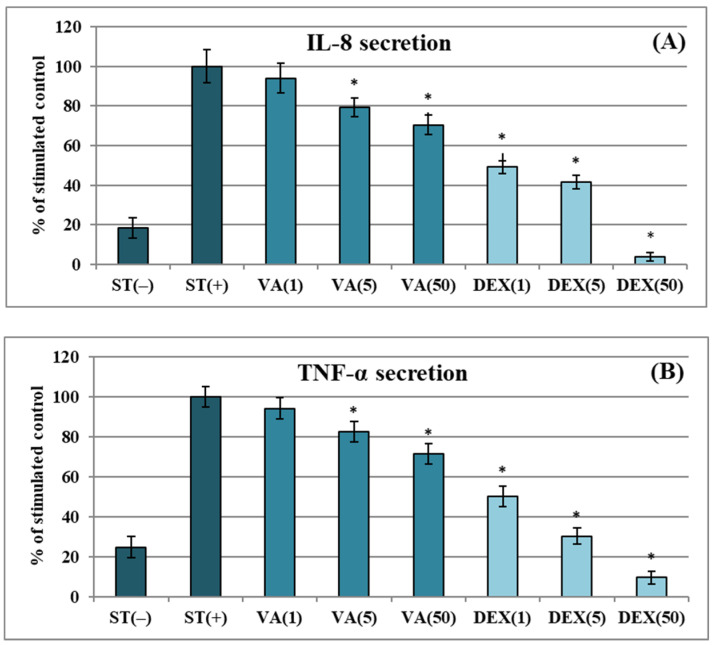
The effect of vanillic acid (VA) at concentrations of 1–50 µg/mL on: (**A**) IL-8 and (**B**) TNF-α secretion by LPS-stimulated neutrophils [%]. Data are mean values ± SD of three independent experiments performed with cells isolated from five independent donors. Positive control: dexamethasone (DEX) at 1–50 µg/mL. Statistical significance of differences (*) was established by ANOVA with post hoc Dunnett’s test, with * *p* < 0.05 compared with the stimulated control.

**Figure 4:**
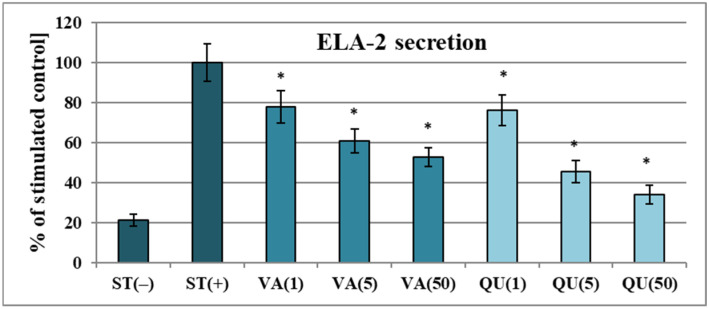
The effect of vanillic acid (VA) at concentrations of 1–50 µg/mL on ELA-2 secretion by *f*MLP+cytochalasin B-stimulated neutrophils [%]. Data are presented as means ± SD of three independent experiments performed with cells isolated from five independent donors. Positive control: quercetin (QU) at 1–50 µg/mL. Statistical significance in Dunnett’s test: * *p* < 0.05 compared with the stimulated control.

The influence of VA on the release of key pro-inflammatory mediators was also investigated in the neutrophil model. As shown in Figure 3A,B, VA moderately suppressed the secretion of cytokines and chemokines in LPS-stimulated cells, with inhibition rates of 20.7–29.6% for IL-8 and 17.4–28.5% for TNF-α at 5–50 µg/mL (*p* < 0.05). In comparison, dexamethasone (DEX, a glycosteroid anti-inflammatory drug used as a positive control) was 2–3 times more potent at the concentration of 5 μg/mL. On the other hand, VA was a very effective inhibitor of ELA-2 secretion from *f*MLP+cytochalasin B-stimulated neutrophils (Figure 4). The inhibition rates for ELA-2 were 22.1%, 39.2%, and 47.5% at 1, 5, and 50 μg/mL of VA, respectively. Noteworthy, VA at 1 μg/mL (*p* > 0.05) reduced the release of the pro-inflammatory enzyme with a potency comparable to a positive control of QU (a flavonol aglycone with well-established anti-ELA-2 effects) and only demonstrated 1.3–1.4 times weaker activity than QU at 5–50 μg/mL. Compared to chlorogenic acid previously studied in the same model [6], VA (at 50 μg/mL, *p* > 0.05) showed a similar ability to downregulate ELA-2 and IL-8, while its effect toward TNF-α was 1.3-fold weaker.

All these data indicated the noticeable capacity of VA to modulate the pro-inflammatory functions of human neutrophils. Moreover, although previous studies of VA in immune cell models have been limited to isolated tests in animal cells, their results generally align with ours. This reinforces the anti-inflammatory potential of VA in immune cells. Specifically, it has been demonstrated that VA at concentrations of 10–100 μM (1.7–16.8 µg/mL) downregulates the secretion of TNF-α and IL-6 in LPS-stimulated mouse peritoneal macrophages by up to 30–63% at 100 μM [29] but has no effect on the production of nitrite (NO) in LPS-stimulated RAW 264.7 macrophages, even at levels up to 125 µg/mL [30]. Together with our results, these findings suggest that VA may influence the release of specific pro-inflammatory factors, including the cytokines tested.

The neutrophilic pro-inflammatory factors we investigated play a crucial role in the development of chronic human disorders. As a pleiotropic master cytokine and chief priming agonist of neutrophils, TNF-α coordinates the inflammatory cascades, stimulates gene expression of key pro-inflammatory mediators, and modulates the recruitment and adhesion of immune cells to vascular endothelium, which is a fundamental step in the inflammatory processes [31]. The second factor, neutrophil elastase 2 (ELA-2), is a metalloproteinase degrading the extracellular matrix components, primarily collagen and elastin, thus, weakening the blood vessel walls and increasing tissue permeability to pro-inflammatory mediators [32]. ELA-2 also stimulates the release of some chemoattractants for neutrophils and other granulocytes, including the primary chemotactic cytokine IL-8, thus, amplifying inflammatory responses [25]. Due their essential roles in inflammation, these factors are implicated in numerous chronic health conditions, including diabetes, cardiovascular disease, atherosclerosis, metabolic syndrome, rheumatoid arthritis, airway diseases, and several types of cancer [33].

Given the wide range of chronic human diseases associated with the upregulated pro-oxidant functions of neutrophils and secretion of ELA-2, TNF-α, and IL-8, the results of the present study might indicate the beneficial health effects of dietary VA. This hypothesis is also supported by the recent multicenter prospective cohort studies on the dietary intake of 36 prevalent polyphenols that reported on VA as one of the primary phenolics detected in human plasma, next to caffeic acid, 4-hydroxy-phenylacetic acid, 4-hydroxy-benzoic acid, quercetin, and protocatechuic acid. What is essential is that an increasing plasma level of VA seemed to be strongly associated with proper body weight maintenance, a crucial factor in lowering the risk of chronic disorders, such as metabolic syndrome, diabetes, and cardiovascular disease [34]. Collectively with the results of in vivo animal studies [16,17,18,19,20,21,22,23,24], all the accumulated data support the protective potential of VA against chronic oxidative stress and inflammation-related ailments.

### 2.2. Biocompatibility with Human Immune Cells

**Figure 5:**
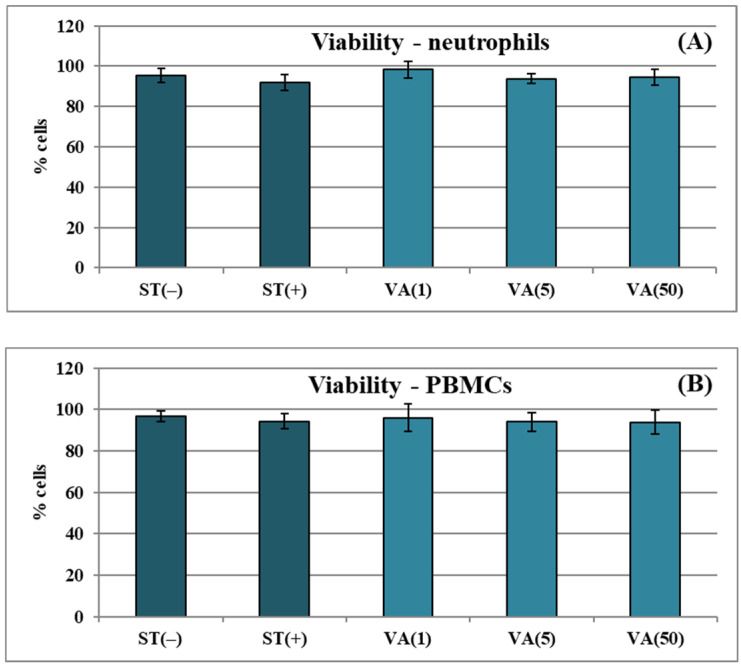
The effect of vanillic acid (VA) at 1–50 µg/mL on membrane integrity of immune cells expressed as a percentage of propidium iodide-negative cells: (**A**) influence on viability of neutrophils after 24 h incubation; (**B**) influence on viability of PBMCs after 48 h incubation. Data are expressed as means ± SD of five independent tests performed with cells isolated from five independent donors. Statistical significance in Dunnett’s test: *p* > 0.05 compared with the stimulated control.

The potential cytotoxicity of VA was examined by flow cytometry and propidium iodide staining on two types of human immune cells ex vivo. The viability of neutrophils and PBMCs incubated with VA at concentrations ranging from 1 to 50 μg/mL was 94.0–98.5% (Figure 5A,B) and did not differ significantly (*p* > 0.05) from that of LPS-stimulated controls (91.7–94.5%) and non-stimulated cells (95.2–97.0%). Therefore, VA might be considered biocompatible with the human immune cells tested.

### 2.3. Antioxidant Activity in Non-Cellular Models

Polyphenols have the ability to reduce ROS levels either directly, by scavenging, or indirectly, by influencing cellular pathways associated with their generation or elimination [35]. Therefore, after confirming the ability of VA to downregulate ROS levels in a neutrophil model, we also examined its direct antioxidant effects on various physiologically significant ROS. The ROS tested included superoxide (O_2_^•−^), hydrogen peroxide (H_2_O_2_), hydroxyl radical (HO^•^), nitric oxide (NO^•^), and hypochlorous acid (HClO), all of which are released by stimulated neutrophils during oxidative burst [28].

As shown in Table 1, VA demonstrated a concentration-dependent scavenging potential against all tested ROS, except for HO^•^, displaying varying effectiveness toward specific oxidants. Compared to reference antioxidants, the most vigorous effects were observed against HOCl and H_2_O_2_. Two positive controls were tested simultaneously: Trolox (TX), a synthetic vitamin E analog widely used for evaluating the antioxidant capacity of food-derived products, and quercetin (QU), one of the most effective phenolic antiradical agents, revealing strong effects in all tested models [36]. Notably, the scavenging capacity of VA for HOCl and H_2_O_2_ was comparable to those of QU and surpassed TX by 11-fold and 1.5-fold, respectively. Moreover, in comparison with a variety of phenolic acids and other phenols previously tested using the same assay protocols [36,37], VA ranked among the most potent scavengers of both HOCl and H_2_O_2_. For instance, literature SC_50_ values for HOCl scavenging varied from 3.15 to 5.62 µg/mL for *p*-coumaric, dihydrocaffeic, and protocatechuic acids and up to 13.80 µg/mL for chlorogenic acid [36,37]. In contrast, this study found an SC_50_ of 1.74 µg/mL for VA (Table 1). Similarly, literature SC_50_ values for H_2_O_2_ scavenging ranged between 5.23 µg/mL for dihydrocaffeic acid and 120.50 µg/mL for *p*-coumaric acid [36,37], while this study observed an SC_50_ of 10.40 µg/mL for VA (Table 1).

As distinguished from its potent reactivity with HOCl and H_2_O_2_, the effects of VA toward O_2_^•−^ and NO^•^ were considerably weaker compared to all phenolic standards tested in this (Table 1) and previous works utilizing the same fluorimetric protocols [36,37]. However, in the fluorimetric studies of Zhao et al. [30], VA had a NO^•^-scavenging capacity similar to QU, with SC_50_ values of 23.38 and 20.44 µg/mL, respectively. This discrepancy in VA effectiveness against NO^•^ might be attributed to differences in assay protocols, such as the use of DMSO and higher reagent concentrations by Zhao et al. [30]. However, a direct comparison of the two protocols is necessary to clarify the source of this variability.

Since O_2_^•−^ is the primary ROS generated by activated immune cells and serves as a precursor to other ROS, the relative effectiveness of VA versus QU observed in the neutrophil and non-cellular tests, primarily the O_2_^•−^ scavenging assay, suggests that the antioxidant effects in the cellular model likely arise from indirect mechanisms rather than direct ROS scavenging. As shown in Figure 1 and Table 1, the antioxidant potency of QU exceeded that of VA by a factor of 41 in direct O_2_^•−^-scavenging, while it only surpassed VA by 2–3 fold in reducing ROS levels in stimulated cells. Nevertheless, while the direct redox activity of VA appears less significant in a biological context, it could enhance the practical value of VA as a food additive, flavoring agent, and food stabilizer. As the relevant literature data are limited, future research should explore this subject thoroughly.

### 2.4. Antioxidant Activity in Human Plasma Model

The pro-oxidant effects of ROS generated by immune cells primarily result from the formation of highly toxic secondary reactive species. A notable example is peroxynitrite (ONOO^−^), an aggressive oxidant and nitrative agent produced in the reaction between O_2_^•−^ and NO^•^. It interacts rapidly with various biomolecules in the body, disrupting their physiological functions by causing critical changes in their structures, which include the nitration and dimerization of tyrosine residues in proteins, the oxidation of protein thiols, and the promotion of protein carbonylation and lipid peroxidation [38].

**Figure 6:**
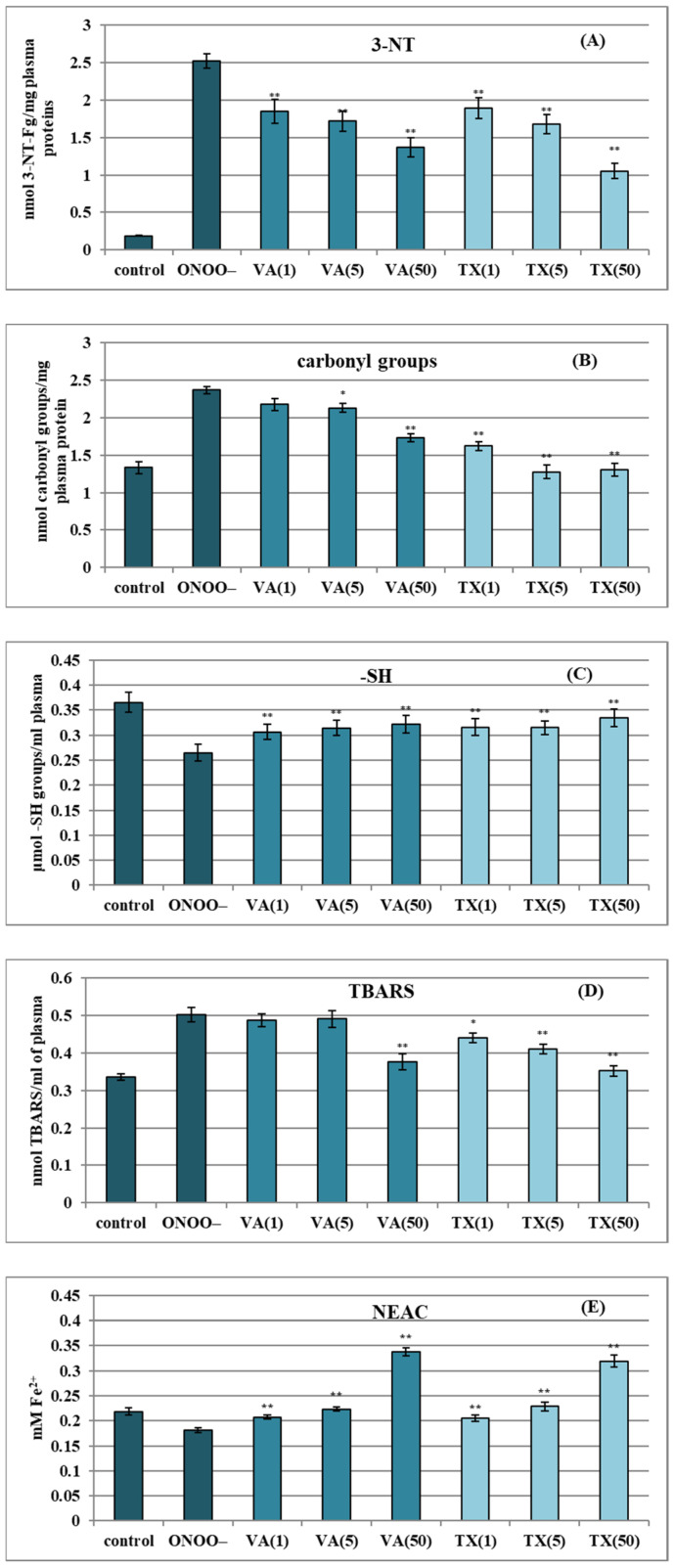
The protective effects of vanillic acid (VA) at 1–50 µg/mL on human plasma under oxidative stress: (**A**) influence on the nitration of tyrosine residues in plasma proteins and formation of 3-nitrotyrosine (3-NT); (**B**) influence on the formation of protein carbonyl groups; (**C**) effects on the level of free thiol groups (-SH) in plasma proteins; (**D**) impact on the lipid peroxidation and levels of thiobarbituric acid-reactive substances (TBARS); (**E**) effects on the non-enzymatic antioxidant capacity (NEAC) of plasma (assessed by FRAP test). Positive control: Trolox^®^ (TX) at 1–50 µg/mL. Data are expressed as mean values ± SE (*n* = 8–12). Statistical significance: * *p* < 0.05 and ** *p* < 0.01, for ONOO^−^-treated plasma in the presence of the examined compounds (1–50 μg/mL) versus ONOO^−^-treated plasma in the absence of the studied substances.

As illustrated in Figure 6, VA demonstrated significant protective potential against damage to human plasma biomolecules exposed to 100–150 μM ONOO^−^. The applied ONOO^−^ levels corresponded to in vivo concentrations that can arise in local compartments during conditions of supraphysiological production of NO^•^ and O_2_^•−^, such as in atherosclerosis or other inflammatory events affecting blood vessels [38].

To assess the plasma antioxidant capacity and the rate of oxidative/nitrative changes to plasma components, we measured levels of well-established biomarkers of protein nitration (3-nitrotyrosine, 3-NT, Figure 6A), modification of amino acid side chains (concentration of carbonyl groups, Figure 6B), oxidation of free thiol groups in proteins (-SH, Figure 6C), lipid peroxidation, and formation of thiobarbituric acid-reactive substances (TBARS) (Figure 6D), as well as the non-enzymatic antioxidant capacity of plasma (NEAC/FRAP, Figure 6E).

The addition of ONOO^−^ to the plasma samples caused a significant (*p* < 0.01) increase in 3-NT (Figure 6A), carbonyl group concentrations in protein side chains (Figure 6B), and TBARS levels (Figure 6D). Meanwhile, the levels of free thiols (Figure 6C) and NEAC values (Figure 6E) decreased significantly. Incubating plasma samples with VA at 1–50 µg/mL resulted in noticeable protection against the damaging impact of ONOO^−^ (*p* < 0.01–0.05), with the most potent effects observed toward 3-NT, thiols, and NEAC values. As shown in Figure 6A, VA reduced tyrosine nitration by 27–32% at 1–5 µg/mL (5.9–29.8 μM; *p* < 0.01) and by 51% at 50 µg/mL (*p* < 0.01). VA also effectively inhibited thiol oxidation (Figure 6C) by 42–50% at 1–50 µg/mL (*p* < 0.01), indicating significant protective effects across a wide range of concentrations. Additionally, VA limited the detrimental impact of ONOO^−^ toward the NEAC plasma value across the tested levels (Figure 6E). The tested compound nearly doubled the reducing potential of blood plasma at 50 μg/mL and restored NEAC to physiological levels (as measured in control plasma not treated with ONOO^−^) at lower concentrations (*p* < 0.01).

Figure 6B illustrates the ability of VA to counteract the formation of carbonyl groups in protein side chains. The ONOO^−^-related damage to proteins was limited by up to 55%, but statistically significant effects were observed only at 5–50 µg/mL (*p* < 0.01–0.05). A similar inhibitory potential of VA was noted regarding lipid peroxidation (Figure 6D); significant protection was detectable only at 50 µg/mL (*p* < 0.01); however, under these conditions, the ONOO^−^-related oxidative modifications of lipids, reflected in rising TBARS levels, were reduced by as much as 83%.

All results obtained in the human plasma model indicated a comparable effectiveness of VA and the reference antioxidant TX. Therefore, the protective effects in plasma could be viewed as one of the synergistic mechanisms behind the health benefits of dietary VA. However, the bioavailability of VA must be taken into consideration by interpreting the results from the plasma model. As demonstrated in previous animal studies, the total plasma levels of VA can reach up to 0.92 µg/mL after oral administration of the pure compound [39] and 1.4 µg/mL following the consumption of a VA-rich herbal preparation [40], with bioavailability estimated to be between 25% and 36% [39]. In humans, long-term supplementation with VA-containing foods has resulted in plasma levels of up to 0.46 µg/mL [41]. These levels are consistent with the VA concentrations of 1–5 µg/mL used in our study, particularly given that acute stress conditions were applied in the model. In contrast, recent cohort studies on polyphenol intake from typical human diets indicate that median VA levels in plasma average 189 nM (31.8 ng/mL) [34]. Based on these findings and our results, it might be concluded that the VA levels associated with a typical diet may not be sufficient to significantly enhance health as a single factor. However, these levels may still be relevant when VA is supplemented with VA-rich foods and when it is considered as part of a complex matrix of dietary phenolics due to the expected additive and synergistic effects. In light of this, the potent effects of VA observed at 5–50 µg/mL in the current study may reflect such relationships. Nonetheless, further in vitro and in vivo research is needed to explore the additive and synergistic potential of VA within complex phenolic matrices.

### 2.5. Influence on Non-Enzymatic Protein Glycation

Glycation is a non-enzymatic reaction between carbonyl groups of reducing sugars and free amino groups of biomolecules, mainly proteins [42]. Due to its pro-oxidant and pro-inflammatory effects, it is associated with numerous human diseases, primarily diabetes and cardiovascular disorders [42,43]. Given that dietary plant antioxidants may provide an effective strategy to prevent glycation [43,44], we aimed to evaluate the anti-glycation potential of VA in the BSA–fructose model. We monitored the impact of VA on the formation of advanced glycation end products (AGEs), which are considered primary contributors to glycation-related toxicity in vivo [42,43,44].

As shown in Table 1, VA demonstrated a robust inhibitory effect on non-enzymatic protein glycation and the formation of AGEs. With an IC_50_ value of 46.4 µg/mL, VA was 1.7 times more potent than aminoguanidine (IC_50_ = 78.9 µg/mL), a clinically tested anti-glycation agent used as a positive control. On the other hand, VA appeared less effective compared to phenolic compounds known for their stronger hydroxylation, which were tested previously in the same model, such as chlorogenic acid or quercetin glycosides (IC_50_ in the range of 3.09–10.90 µg/mL) [37,45].

According to the literature, polyphenols may reduce glycation in non-cellular models by scavenging glycation-triggering ROS, trapping glycation intermediates like reactive dicarbonyls, or binding to proteins, thus, limiting access to amino groups for the reaction [41,43]. Notably, recent in silico docking studies indicated that VA, like other phenolic acids such as caffeic and ferulic acid, has relatively poor binding affinity to BSA when compared to flavonoids and catechins [46]. Furthermore, a direct reaction with glyoxal has demonstrated a similar dicarbonyl-trapping capacity for VA and other phenolic acids, like chlorogenic, ferulic, and gallic acid [47]. In this context, weaker anti-AGE effects of VA (as shown in Table 1) compared to those reported earlier for both quercetin derivatives and chlorogenic acid [37,45] appear to largely stem from their differing abilities to act through the third potential antiglycation mechanism: ROS scavenging. We indeed noted a dramatic difference between VA and QU (Table 1), as well as previously studied polyphenols [37,45] in their capacity to scavenge O_2_^•−^, the primary ROS generated during glycation [48].

Despite this, the relatively potent ability of VA to inhibit the formation of AGEs compared to aminoguanidine still suggests its biological relevance. It aligns also with existing experimental data from biological systems. For instance, VA has been reported to effectively protect Neuro-2A cells against glycation-mediated toxicity [49] and inhibit AGE formation in processed meat [50]. Nevertheless, further studies are needed to verify the VA potential as an anti-glycation agent in vivo and to understand all possible mechanisms of VA activity related to glycation chemistry and glycation-associated metabolic pathways.

## 3. Materials and Methods

### 3.1. Biocompatibility and Bioactivity of VA in Human Neutrophils and PBMCs Models

Neutrophils and PBMCs were isolated from human buffy coats by dextran sedimentation, erythrocyte lysis, and centrifugation in a Ficoll-Hypaque gradient (PAA Laboratories, Pasching, Austria), as previously described [51,52]. The buffy coats were purchased from the Regional Centre of Blood Donation and Blood Treatment in Lodz (Poland). Peripheral venous blood for fractionation and preparation of buffy coats was collected in the Centre from healthy male volunteers (ages 18–35 years) who were not taking any medications. The study was conducted following the Declaration of Helsinki and approved by the Committee on the Ethics of Research at the University of Lodz (8/KBBN-UŁ/II/2015) and by the Committee on the Ethics of Research at the Medical University of Lodz (RNN/15/23/KE).

The inhibition of ROS production by neutrophils stimulated with *f*MLP (*N*-formyl-methionyl-leucyl-phenyl-alanine) was analyzed using a luminol-amplified chemiluminescence assay [51]. The impact on the release of neutrophil elastase (ELA-2) by *f*MLP+cytochalasin B-stimulated cells was tested spectrophotometrically for the level of *p*-nitrophenol formed from *N*-succinyl-alanine-alanine-valine-*p*-nitroanilide (SAAVNA) as a precursor [6]. The secretion of TNF-α and IL-8 by lipopolysaccharide (LPS)-stimulated neutrophils was tested using ELISA tests following the instructions. The effect on ROS, ELA-2, and cytokine production was calculated as a percentage of the released markers compared to the control cell samples untreated by VA (100% activity). Dexamethasone (DEX) and quercetin (QU) were positive controls. Control cell samples that contained no analytes, positive controls, and stimulants were also prepared. All analytes (VA, DEX, and QU) were tested at 1–50 μg/mL. LPS (from *Escherichia coli* O111:B4) was purchased from Merck Millipore (Billerica, MA, USA), ELISA tests from BD Biosciences (San Jose, CA, USA), and all other reagents from Sigma-Aldrich (St. Louis, MO, USA). All measurements were performed in 96-well plates using a microplate reader (SYNERGY 4, BioTek, Winooski, VT, USA).

The cellular biocompatibility of VA toward neutrophils and PBMCs was assessed by flow cytometry (BD FACSCalibur, BD Biosciences, San Jose, CA, USA) with propidium iodide (PI) staining and Triton X-100 solution as a positive control [51]. VA was tested at 1–50 μg/mL. The analyses were performed after 24 h and 48 h incubation for neutrophils and PBMCs, respectively.

In the tests in the human neutrophil model, control samples were prepared from cells not treated with extracts and/or stimulating factors. In experiments with cells and extracts alone, no pro-oxidant or pro-inflammatory effect was observed. Moreover, reaction controls were implemented to ensure that all measurements worked correctly and that VA did not interfere with any of the reagents.

### 3.2. Protective Effects on Human Plasma Components

Blood plasma was isolated by differential centrifugation of buffy coats from healthy volunteers, as previously described [45]. The buffy coat fractions (by-products of blood fractionations for transfusions) were obtained from the Regional Centre of Blood Donation and Blood Treatment in Lodz (Poland), where they were collected from adult donors (over 18 years old). The donors were clinically recognized as healthy (meeting the criteria of being a blood donor for people needing a transfusion) and not taking medications such as antibiotics, anti-inflammatory agents, or antihistamines. On the blood donation day, the donors were only allowed to eat a low-fat meal. For a few days before donating blood, donors must limit smoking and not drink alcohol. The study conformed to the principles of the Declaration of Helsinki.

For the tests, plasma samples were pre-incubated for 15 min at 37 °C with VA or positive control (Trolox^®^, TX, Sigma-Aldrich, St. Louis, MO, USA) and then treated with ONOO^−^ (100–150 μM). Two kinds of control plasma were also prepared; the first was exposed to ONOO^−^ without the analytes, and the second was the blind control containing neither analytes nor ONOO^−^. The protective effect of the tested analytes on human plasma components under the conditions of oxidative stress induced by ONOO^−^ was verified spectro/fluorimetrically and immunoenzymatically, as previously described [53,54,55]. In brief, the non-enzymatic antioxidant capacity (NEAC) of plasma was measured in the FRAP assay, based on the reducing ferric ions (Fe^3+^) into ferrous ions (Fe^2+^); the results were expressed in mM of Fe^2+^ equivalents. A competitive ELISA test was applied to the immunodetection of 3-nitrotyrosine (3-NT). All results from this assay were expressed as equivalents of a 3-nitrotyrosine-containing protein standard (nitrated fibrinogen, 3-NT-FG) per mg of blood plasma protein. The concentration of free thiol (-SH) groups in plasma was measured spectrophotometrically using Ellman’s reagent and expressed in µmol/mL of plasma. The peroxidation of plasma lipids was assayed using thiobarbituric acid-reactive substances (TBARS), calculated in nmol/mL of plasma.

The carbonyl groups were detected in plasma proteins using a modified protocol of [56]. Briefly, blood plasma samples were diluted to obtain a final concentration of 5 μg of protein/mL, layered into microplate wells (200 μL), and incubated for a night at 4 °C. Then, the plate was washed three times with 0.02 M phosphate-buffered saline (PBS, 3 × 250 μL/well) and incubated with the DNPH solution (0.05 mM, pH 6.2; 200 μL/well, for 45 min, in the dark). The DNPH solution was then removed by washing (5 × 250 μL/well) with the 0.02 M PBS/ethanol mixture (1:1; *v*/*v*). The next step was blocking the well surface with defatted milk solution (5%, in 0.02 M PBS; 250 μL/well) for 1.5 h at 37 °C. After removing the milk solution, microplate wells were washed with 0.02 M PBS enriched with 0.01% Tween 20 (PBST; 3 × 250 μL/well). For the carbonyl detection, the anti-DNP antibody solution (1:2500) was added (200 μL/well). The 1 h incubation (at 37 °C) was followed by washing with PBST (250 μL/well) 5 times. Next, the second antibody solution (1:2500; conjugated with the horseradish peroxidase) was added (200 μL/well), and the plate was incubated for 1 h at 37 °C. After the incubation, unbound antibodies were removed by washing with PBST (250 μL/well) 5 times. The reaction was visualized using a SIGMAFAST™ OPD (*o*-phenylenediamine dihydrochloride) solution (200 μL/well) and stopped with 50 μL of 40% sulphuric acid. The absorbance was recorded at 490 nm, and the results were calculated based on a standard curve prepared from the oxidized and reduced albumin. All measurements were performed in 96-well plates using a microplate reader (SYNERGY 4, BioTek, Winooski, VT, USA). All reagents were from Sigma-Aldrich (St. Louis, MO, USA).

In the tests in the human plasma model, control samples were prepared with plasma untreated with the extracts and/or ONOO^−^. In the experiments with blood plasma and the extracts only (without adding ONOO^−^), no pro-oxidative effect was found. Moreover, reaction controls were implemented to ensure that all measurements worked correctly and that VA did not interfere with any of the reagents.

### 3.3. Inhibition of Non-Enzymatic Protein Glycation

The influence of VA on non-enzymatic protein glycation was tested by measuring the level of advanced glycation end products (AGEs) in the milieu of bovine serum albumin (BSA) and fructose using the modified fluorimetric method adapted from [45]. The tests were carried out at a minimum of 5 concentration levels of VA, with the IC_50_ value (half inhibitory concentration) assessed. The blank samples of BSA with and without adding VA were used to compensate the background fluorescence of the reagents. The positive control was aminoguanidine (AG). All measurements were performed in 96-well plates using a multilabel counter Victor 1420 (Perkin Elmer Life and Analytical Sciences, Shelton, CT, USA).

### 3.4. Scavenging Activity Against Multiple Oxidants

The studies were conducted using modified in vitro spectrophotometric and fluorimetric methods described by [36]. TX was employed as a positive control in all tests and a series of reaction controls were implemented to ensure that all measurements worked correctly and that VA did not interfere with any of the reagents except for the target oxidants.

The superoxide anion (O_2_^•−^)-scavenging capacity was assessed by measuring the concentration of formazan produced by reducing nitrotetrazolium blue (NBT) with O_2_^•−^ generated in the reaction of xanthine oxidase with xanthine. Analyses of control samples confirmed that VA did not interfere with either NBT or xanthine; moreover, tests assessing uric acid production by the enzyme ruled out the direct inhibition of xanthine oxidase by VA [57].

The hydrogen peroxide (H_2_O_2_)-reducing potential was examined by monitoring the formation of quinoneimine in the reaction involving phenol, 4-aminoantipyrine (4-AAP) and H_2_O_2_, catalyzed by horseradish peroxidase (HRP). Blank samples containing H_2_O_2_, 4-AAP, and HRP, with and without VA, were tested to account for the background absorbance of reagents and determine whether VA interfered with the measurements. Additionally, to assess if VA could inhibit HRP directly, HRP was pre-incubated with VA for 5 min before the addition of H_2_O_2_ [58]; the tests confirmed that VA influenced quinoneimine formation solely by scavenging H_2_O_2_.

Nitric oxide (NO^•^) scavenging was assessed by measuring the fluorescence of triazofluorescein (DAF-2T), formed in the reaction between 4,5-diaminefluorescein (DAF-2) with NO^•^, which was spontaneously released from sodium nitroprusside (SNP) as a donor. Analyses of control samples confirmed that VA did not interfere with SNP, DAF-2, or DAF-2T.

The hypochlorous acid (HOCl)-scavenging potential was determined by assessing the level of 5,5-dithiobis-2-nitrobenzoic acid (DTNB), a product of the oxidation of 5-thio-2-nitrobenzoic acid (TNB) in the presence of HOCl (generated from sodium hypochlorite, NaOCl). To confirm that VA did not react with DTNB or TNB, a 5 min pre-incubation of VA with HOCl was applied before adding TNB for the final incubation.

The ability to scavenge hydroxyl radical (HO^•^) was analyzed by evaluating formation of dihydroxybenzoic acids in the reaction of salicylic acid with HO^•^, generated from the reaction of H_2_O_2_ and Fe^2+^ ions. Control samples containing H_2_O_2_ and Fe^2+^ ions, with or without VA, were analyzed to adjust for the background absorbance of the reagents and exclude any interference from VA.

All measurements were performed in 96-well plates using a multilabel counter Victor 1420 (Perkin Elmer Life and Analytical Sciences, Shelton, CT, USA) or microplate readers SPECTROstar Nano (BMG LabTech, Ortenberg, Germany). 

### 3.5. Statistical Analysis

All samples were assayed in replicates, and results are displayed as the mean values ± SD (standard deviation) or ±SE (standard error) for the indicated number of experiments. The statistical significance of differences between the means was calculated using one-way analysis of variance (ANOVA) or one-way ANOVA for repeated measures, followed by post hoc Tukey’s test for multiple comparisons or post hoc Dunnett’s test. All calculations were performed using the software Statistica 13.1 Pl (StatSoft, Krakow, Poland).

## 4. Conclusions

Our findings reveal, for the first time, the antioxidant and anti-inflammatory potential of VA in human immune cells and plasma. We demonstrated that this dietary phenolic acid is biocompatible with human neutrophils and PBMCs ex vivo across a wide range of 1–50 µg/mL. Furthermore, VA at 5–50 µg/mL significantly reduces the levels of reactive oxygen species (ROS) generated during the oxidative burst of stimulated neutrophils and markedly downregulates the release of the tissue-remodeling enzyme ELA-2 at 1–50 µg/mL. Conversely, its effect on the secretion of pro-inflammatory cytokines, such as TNF-α and IL-8, in stimulated human neutrophils is less pronounced, although it remains statistically significant at 5–50 µg/mL. Additionally, VA at 1–50 µg/mL effectively enhances the non-enzymatic antioxidant capacity of human plasma in vitro and prevents oxidative and nitrative damage to plasma proteins, primarily by safeguarding tyrosine moieties and thiols from the harmful effects of ONOO^−^, a model for circulating ROS. VA also acts as a robust inhibitor of non-enzymatic protein glycation in vitro and the formation of AGEs, showing a stronger potency than aminoguanidine. Together with existing pharmacokinetic data from animal models and studies on polyphenol intake in humans, our results indicate that VA might be recognized as one of the active phenolic components contributing to the dietary protective effects of VA-rich food products against oxidative stress and inflammation-related diseases. On the other hand, our results suggest that the relatively low levels of VA in human plasma associated with a typical diet may not be sufficient to significantly enhance health as a single factor. Nonetheless, these levels may still be relevant when VA is considered as part of a complex matrix of dietary phenolics. Further mechanistic studies involving a broader range of normal human cells, as well as human intervention trials, are essential to validate the health benefits of VA and to explore its additive and synergistic potential within complex phenolic matrices.

## Figures and Tables

**Table 1:** Scavenging activity (SC_50_) towards multiple oxidants and inhibitory potential (IC_50_) against protein glycation.

Target Factor	Vanillic Acid (VA) (µg/mL)	Reference Standards		
		Quercetin (QU) (µg/mL)	Trolox (TX) (µg/mL)	Aminoguanidine (µg/mL)
NO^•^	12.4 ± 0.93 ^B^	0.48 ± 0.03 ^A^	0.59 ± 0.02 ^A^	-
HOCl	1.74 ± 0.01 ^A^	2.08 ± 0.14 ^A^	19.8 ± 0.44 ^B^	-
O_2_^•−^	326 ± 29.9 ^C^	7.89 ± 0.24 ^A^	139 ± 2.99 ^B^	-
H_2_O_2_	10.4 ± 0.33 ^B^	8.00 ± 1.01 ^A^	16.1 ± 0.49 ^C^	-
HO^•^	>500	51.3 ± 2.53 ^A^	129 ± 2.35 ^B^	-
AGE formation	46.4 ± 1.47 ^A^	-	-	78.9 ± 4.95 ^B^

SC_50_, half scavenging concentration (amount required to reduce the initial oxidant concentration by 50%) and IC_50_, half inhibitory concentration (amount needed for 50% inhibition of AGE formation) calculated in µg/mL. Data are given as means of three replicates ± SD. Values in each row labeled with different capital letters (A–C) are significantly different with *p* < 0.05.

## Data Availability

Data are contained within the article.

## Abbreviations

3-NT	3-nitrotyrosine
AG	aminoguanidine
AGEs	advanced glycation end products
AP-1	activator protein 1
BSA	bovine serum albumin
DEX	dexamethasone
DNPH	2,4-dinitrophenylhydrazine
ELA-2	enzyme elastase-2
eNOS	endothelial nitric oxide synthase
*f*MLP	*N*-formyl-methionyl-leucyl-phenyl-alanine
H_2_O_2_	hydrogen peroxide
HClO	hypochlorous acid
HO^•^	hydroxyl radical
IL	interleukin
LPS	lipopolysaccharide
NBT	nitrotetrazolium blue
NEAC	non-enzymatic antioxidant capacity of human plasma
NF-κB	nuclear factor kappa-light-chain-enhancer of activated B cells
NO^•^	nitric oxide
ONOO^−^	peroxynitrite
O_2_^•−^	superoxide
PI	propidium iodide
PBMCs	peripheral blood mononuclear cells
QU	quercetin
RAGE	receptor for advanced glycation endproducts
ROS	reactive oxygen species
SAAVNA	*N*-succinyl-alanine-alanine-valine-*p*-nitroanilide
SH-	free thiol groups
ST(+)/ST(−)	stimulated control/unstimulated control
TBARS	thiobarbituric acid-reactive substances
TFPI	tissue factor pathway inhibitor
TNF-α	tumor necrosis factor α
TX	Trolox^®^
VA	vanillic acid
